# Real-world safety and effectiveness of inhaled nitric oxide therapy for pulmonary hypertension during the perioperative period of cardiac surgery: a post-marketing study of 2817 patients in Japan

**DOI:** 10.1007/s11748-023-01971-2

**Published:** 2023-09-15

**Authors:** Emi Matsugi, Shigeki Takashima, Shuhei Doteguchi, Tomomi Kobayashi, Motohiro Okayasu

**Affiliations:** 1 Medical Affairs, Mallinckrodt Pharmaceuticals, 1-12-32 Akasaka, Minato-ku, Tokyo, 107-6030 Japan; 2 Pharmacovigilance, Mallinckrodt Pharmaceuticals, Tokyo, Japan

**Keywords:** Cardiac surgery, Inhaled nitric oxide, Perioperative, Post-marketing study, Pulmonary hypertension

## Abstract

**Objective:**

To evaluate the real-world safety and effectiveness of inhaled nitric oxide (INOflo^®^ for Inhalation 800 ppm) for perioperative pulmonary hypertension associated with cardiac surgery in Japan.

**Methods:**

This was a prospective, non-interventional, all-case, post-marketing study of pediatric and adult patients who received perioperative INOflo with cardiac surgery from November 2015–December 2020. Safety and effectiveness were monitored from INOflo initiation to 48 h after treatment completion or withdrawal. Safety outcomes included adverse drug reactions, blood methemoglobin concentrations, and inspired nitrogen dioxide concentrations over time. Effectiveness outcomes included changes in central venous pressure among pediatrics, mean pulmonary arterial pressure among adults, and the partial pressure of arterial oxygen/fraction of inspired oxygen ratio (PaO_2_/FiO_2_) in both populations.

**Results:**

The safety analysis population included 2,817 Japanese patients registered from 253 clinical sites (pediatrics, n = 1375; adults, n = 1442). INOflo was generally well tolerated; 15 and 20 adverse drug reactions were reported in 14 pediatrics (1.0%) and 18 adults (1.2%), respectively. No clinically significant elevations in blood methemoglobin and inspired nitrogen dioxide concentrations were observed. INOflo treatment was associated with significant reductions in both central venous pressure among pediatrics and mean pulmonary arterial pressure among adults, and significant improvements in PaO_2_/FiO_2_ among pediatrics and adults with PaO_2_/FiO_2_ ≤ 200 at baseline.

**Conclusions:**

Perioperative INOflo treatment was a safe and effective strategy to improve hemodynamics and oxygenation in patients with pulmonary hypertension during cardiac surgery. These data support the use of INOflo for this indication in Japanese clinical practice.

**Supplementary Information:**

The online version contains supplementary material available at 10.1007/s11748-023-01971-2.

## Introduction

Inhaled nitric oxide (iNO), a selective pulmonary vasodilator, is an effective perioperative strategy for the management of pulmonary hypertension, a potentially life-threatening complication in patients undergoing cardiac surgery with cardiopulmonary bypass (CPB) [1–3]. Given that the vasodilatory effects of perioperative iNO therapy are largely limited to the pulmonary artery, it has the advantage of being able to reduce pulmonary artery pressure without lowering systemic blood pressure (SBP), and can facilitate safe withdrawal from CPB, adjunctive inotropic support, and mechanical ventilation [4]. The utility of iNO for the perioperative management of patients with pulmonary hypertension undergoing cardiac surgery has been recognized in European and American clinical guidelines [5–7], and it is recommended as an adjunctive treatment for patients receiving implantable left ventricular assist devices for advanced heart failure in Japan [8].

Since 2008, INOflo^®^ for Inhalation 800 ppm (medicinal gas containing NO 800 ppm; Mallinckrodt Pharmaceuticals) has been approved in Japan for the improvement of neonatal hypoxic respiratory failure with persistent pulmonary hypertension [9, 10]. In 2011, the Japanese Society of Thoracic Surgery, Japanese Society of Cardiac Surgery, and Japanese Society of Pediatric Cardiology submitted a request to expand the approval of INOflo for the improvement of perioperative pulmonary hypertension associated with cardiac surgery, in line with its approved indication in Europe [11]. To confirm that the dosage and administration approved in Europe was safe and effective in Japanese patients, a small phase 3 study of perioperative INOflo treatment was conducted in 12 pediatric and six adult patients with pulmonary hypertension during cardiac surgery [10, 12]. Based on these positive results, the Japanese Ministry of Health, Labour and Welfare conditionally approved INOflo for the improvement of perioperative pulmonary hypertension associated with cardiac surgery on August 24, 2015 [9, 10, 13].

Given the small sample size of the Japanese phase 3 study, the expanded approval of INOflo was conditional upon further post-marketing surveillance to collect additional safety and effectiveness data in this indication. Herein, we report the results of a large post-marketing study, which aimed to evaluate the real-world safety and effectiveness of INOflo during the perioperative period of cardiac surgery in Japanese patients with pulmonary hypertension.

## Patients and methods

### Study design

This was a prospective, non-interventional, all-case, post-marketing study to monitor the safety and effectiveness of INOflo following its conditional approval for perioperative pulmonary hypertension associated with cardiac surgery in pediatric and adult patients. Eligible patients from 253 clinical sites in Japan were enrolled through anonymized registration forms submitted to a central registration system. The study commenced in November 2015 and eligible patients were analyzed until the target sample size was reached in December 2020. Safety and effectiveness outcomes were monitored from INOflo initiation to 48 h after completion or withdrawal if clinically necessary, or for up to 30 days in patients who required unplanned mechanical support and/or rescue medication to wean from INOflo treatment. This study was reviewed and approved by Japan’s Pharmaceuticals and Medical Devices Agency before commencement, and was conducted in compliance with the Japanese Pharmaceutical and Medical Device Act and Ministry of Health and Welfare Ordinance on Good Post-Marketing Study Practice. Under these regulations, the study was conducted with institutional review board or ethics committee approval from participating sites; informed consent was not required.

### Study population

Enrolled patients were pediatrics (aged < 15 years) and adults (aged ≥ 15 years) who received INOflo for pre-, intra-, or postoperative pulmonary hypertension in conjunction with cardiac surgery after its approval for this indication on August 24, 2015. Consistent with its labeled dosage and administration, pediatric patients received INOflo at an initial dose of 10 ppm, which could be increased to ≤ 20 ppm per physician discretion, and adult patients received INOflo at an initial dose of 20 ppm, which could be increased to ≤ 40 ppm per physician discretion [10]. Treatment with INOflo was maintained until the patient’s hemodynamic and oxygenation status had resolved, which was typically ≤ 7 days. As hemodynamic and oxygenation improved, the INOflo dose was lowered to 5 ppm and inhalation was continued until safe weaning was achievable.

### Outcome measures

Clinical laboratory data were collected before INOflo initiation (i.e., baseline), at the start of treatment, 1–4, 24, and 48 h after INOflo initiation, at treatment completion/withdrawal (prior to weaning), and ≤ 48 h after completion/withdrawal. Safety was assessed by monitoring the incidence of adverse drug reactions (ADRs), blood methemoglobin (MetHb) concentrations, and inspired nitrogen dioxide (NO_2_) concentrations over time. ADRs were coded using the Japanese version of Medical Dictionary for Regulatory Activities Terminology thesaurus terms (MedDRA/J, version 24.1).

The primary effectiveness endpoints in pediatric patients were changes in CVP and PaO_2_/FiO_2_ from baseline at 24 h after INOflo initiation and at the completion/withdrawal of treatment. In adults, the primary effectiveness endpoints were changes in mPAP and PaO_2_/FiO_2_ from baseline at 24 h after INOflo initiation and at treatment completion/withdrawal. Changes in mPAP among pediatrics and CVP among adults were also evaluated for completeness. Secondary effectiveness endpoints in both populations included changes from baseline in mean SBP (mSBP), pulmonary capillary wedge pressure (PCWP), pulmonary vascular resistance, cardiac output, and percutaneous arterial oxygen saturation (SpO_2_). Physicians also evaluated the overall effectiveness of INOflo by grading patients as “markedly improved”, “improved”, “mildly improved”, “unchanged”, or “worsened”, based on changes in hemodynamic parameters.

### Statistical analysis

A target sample of size of 2000 patients (i.e., 1000 pediatrics and 1000 adults) was estimated to provide > 95% statistical power to detect at least one patient with a significant ADR occurring at a frequency of 0.3%. Patients were continuously registered into the study, with the aim of collecting sufficient data to understand the background profile of patients receiving INOflo in clinical practice, verifying the real-world safety and effectiveness of INOflo, and meeting the conditions of the Pharmaceutical Medical Device Act approval for INOflo in Japan.

The safety analysis population included all patients who received INOflo and for whom case report forms (CRFs) were collected. Safety data are expressed as descriptive summaries (e.g., frequencies, incidence rates, means, and standard deviations [SDs] as appropriate) for pediatric and adult patients separately. Patients in the safety analysis population who received industrial NO gas during the treatment period and/or received INOflo for an off-label indication were excluded from effectiveness analyses. Effectiveness results are summarized using means, SDs, and/or 95% confidence intervals (CIs) for pediatric and adult patients separately. Safety and effectiveness analyses were based on observed data, with no imputation for missing values. Where applicable, statistical analyses were performed using Fisher’s exact test or paired t-test, with a significance level of α = 0.05 in two-tailed tests. Statistical analyses were conducted using SAS version 9.4, Microsoft Access 2016, and Microsoft Excel 2016.

## Results

### Study population

**Fig. 1:**
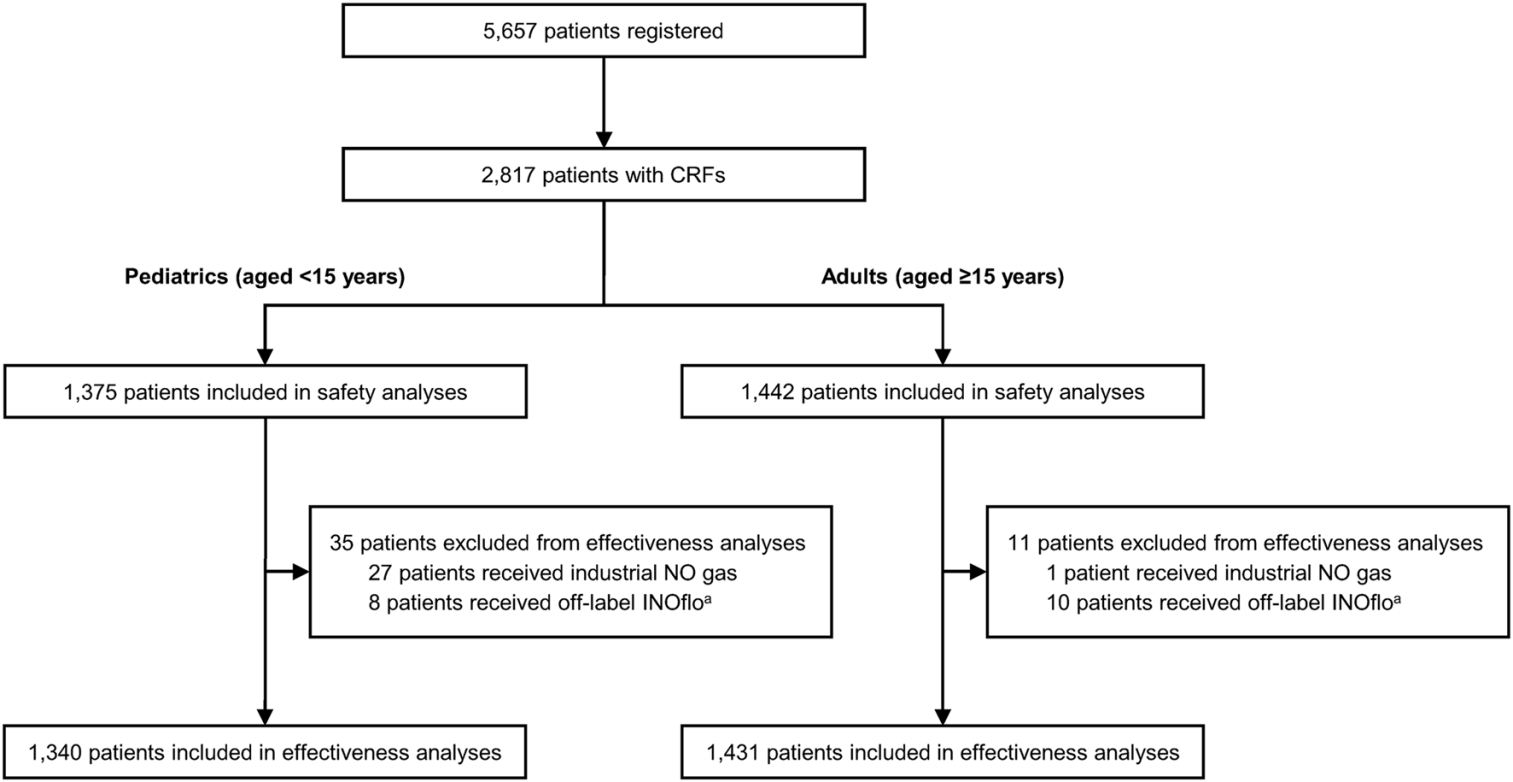
Patient flow diagram, ^a^Patients were excluded if they received perioperative INOflo during procedures other than cardiac surgery, or if cardiac surgery could not be ascertained from case reports forms. *CRF* case report form, *NO* nitric oxide

In total, 5,657 Japanese patients received INOflo for pulmonary hypertension during the pre-, intra-, or postoperative period of cardiac surgery and were registered in this study from November 2015 to March 2019 (Fig. 1). CRFs were collected for 2,817 registered patients who received INOflo, and these patients were subsequently included in the safety analysis population (pediatrics, n = 1375; adults, n = 1442). After excluding patients who received industrial NO gas and/or off-label INOflo treatment, 1,340 pediatrics and 1,431 adults were included in effectiveness analyses.

At baseline, most of the pediatric patients (1,313 of 1,375; 95.5%) were aged < 5 years, with 965 patients (70.2%) aged < 1 year (Table 1). Among the adult patients, 824 of 1,442 (57.1%) were aged ≥ 65 years. More than half of all pediatrics and adults received postoperative INOflo treatment (59.5% and 58.3%, respectively), while 26.8% and 24.1% of patients, respectively, received INOflo during cardiac surgery (Table 1). At baseline, CVP was ≥ 15 mmHg in 172 pediatrics (12.5%) and 324 adults (22.5%), mPAP was ≥ 25 mmHg in 62 pediatrics (4.5%) and 549 adults (38.1%), PaO_2_/FiO_2_ was ≤ 200 in 542 pediatrics (39.4%) and 374 adults (25.9%), and the mPAP/mSBP ratio (Pp/Ps) was > 0.4 in 85 pediatrics (6.2%) and 361 adults (25.0%; Table 1).

**Table 1:** Baseline patient characteristics (safety analysis population)

	Pediatrics (n = 1375)	Adults (n = 1442)
Sex, n (%)		
Male	771 (56.1)	861 (59.7)
Female	604 (43.9)	580 (40.2)
Unknown	0	1 (0.1)
Age, n (%)		
Neonate (≤ 28 days after birth)	295 (21.5)	–
Neonate to < 1 year	670 (48.7)	–
≥ 1 to < 5 years	348 (25.3)	–
≥ 5 to < 10 years	26 (1.9)	–
≥ 10 to < 15 years	23 (1.7)	–
≥ 15 to < 25 years	–	41 (2.8)
≥ 25 to < 35 years	–	69 (4.8)
≥ 35 to < 45 years	–	104 (7.2)
≥ 45 to < 55 years	–	161 (11.2)
≥ 55 to < 65 years	–	242 (16.8)
≥ 65 years	–	824 (57.1)
Unknown	13 (0.9)	1 (0.1)
Presence of pulmonary hypoplasia or history of severe lung disorder, n (%)		
Yes	68 (4.9)	80 (5.5)
No	1280 (93.1)	1332 (92.4)
Unknown	27 (2.0)	30 (2.1)
Timing of INOflo treatment, n (%)		
Before cardiac surgery	20 (1.5)	9 (0.6)
During cardiac surgery	368 (26.8)	347 (24.1)
After cardiac surgery	818 (59.5)	840 (58.3)
Before and during cardiac surgery	2 (0.1)	0
Before and after cardiac surgery	7 (0.5)	3 (0.2)
During and after cardiac surgery	113 (8.2)	211 (14.6)
Before, during, and after cardiac surgery	3 (0.2)	2 (0.1)
Other	15 (1.1)	13 (0.9)
Unknown	29 (2.1)	17 (1.2)
Presence of complications, n (%)		
Yes	472 (34.3)	506 (35.1)
No	903 (65.7)	936 (64.9)
Presence of concomitant drugs, n (%)		
Yes	1292 (94.0)	1321 (91.6)
No	83 (6.0)	121 (8.4)
Baseline mPAP, n (%)		
< 25 mmHg	160 (11.6)	406 (28.2)
≥ 25 to < 50 mmHg	51 (3.7)	522 (36.2)
≥ 50 to < 75 mmHg	10 (0.7)	26 (1.8)
≥ 75 mmHg	1 (0.1)	1 (0.1)
Unknown	1153 (83.9)	487 (33.8)
Baseline CVP, n (%)		
< 15 mmHg	896 (65.2)	785 (54.4)
≥ 15 mmHg	172 (12.5)	324 (22.5)
Unknown	307 (22.3)	333 (23.1)
Baseline PaO_2_/FiO_2_ ratio, n (%)		
≤ 200	542 (39.4)	374 (25.9)
> 200 to ≤ 400	306 (22.3)	365 (25.3)
> 400	167 (12.1)	206 (14.3)
Unknown	360 (26.2)	497 (34.5)
Baseline Pp/Ps ratio, n (%)		
≤ 0.2	30 (2.2)	67 (4.6)
> 0.2 to ≤ 0.4	101 (7.3)	461 (32.0)
> 0.4 to ≤ 0.6	46 (3.3)	277 (19.2)
> 0.6 to ≤ 0.8	21 (1.5)	65 (4.5)
> 0.8 to ≤ 1.0	14 (1.0)	12 (0.8)
> 1.0	4 (0.3)	7 (0.5)
Unknown	1159 (84.3)	553 (38.3)

*CVP* central venous pressure, *FiO*_*2*_ fraction of inspired oxygen, *mPAP* mean pulmonary arterial pressure, *PaO*_*2*_ partial pressure of arterial oxygen, *Pp/Ps* mean pulmonary arterial pressure/mean systemic blood pressure

### Safety outcomes

In total, 15 ADRs were reported in 14 pediatric patients (1.02%) and 20 ADRs were reported in 18 adults (1.25%) during the observation period (Table 2). ADRs with an incidence of ≥ 0.1% in pediatrics were acute kidney injury (four patients; 0.29%), renal failure (two patients; 0.15%), and methemoglobinemia (two patients; 0.15%); and in adults were pulmonary hypertension (eight patients; 0.55%) and pulmonary arterial hypertension (two patients; 0.14%).

**Table 2:** Summary of adverse drug reactions (safety analysis population)

ADR preferred term	Pediatrics (n = 1375)			Adults (n = 1442)		
	Patients with ≥ 1 ADR, n (%)^a^	Serious ADR, n	Non-serious ADR, n	Patients with ≥ 1 ADR, n (%)^a^	Serious ADR, n	Non-serious ADR, n
Total ADRs	14 (1.02)	8	7	18 (1.25)	13	7
Blood and lymphatic system disorders						
Hemorrhagic diathesis	0	0	0	1 (0.07)	0	1
Methemoglobinemia	2 (0.15)	0	2	1 (0.07)	0	1
Cardiac disorders						
Atrial flutter	0	0	0	1 (0.07)	0	1
Metabolism and nutrition disorders						
Hyperglycemia	1 (0.07)	0	1	0	0	0
Vascular disorders						
Hypotension	0	0	0	1 (0.07)	1	0
Respiratory, thoracic, and mediastinal disorders						
Acute respiratory distress syndrome	0	0	0	1 (0.07)	1	0
Bronchial hemorrhage^b^	1 (0.07)	0	2	0	0	0
Hypoxia	1 (0.07)	1	0	0	0	0
Pneumothorax	1 (0.07)	1	0	0	0	0
Pulmonary alveolar hemorrhage	0	0	0	1 (0.07)	1	0
Pulmonary arterial hypertension	0	0	0	2 (0.14)	2	0
Pulmonary hypertension	0	0	0	8 (0.55)	5	4
Pulmonary hypertensive crisis	0	0	0	1 (0.07)	1	0
Renal and urinary disorders						
Acute kidney injury^b^	4 (0.29)	4	0	1 (0.07)	1	0
Oliguria^b^	1 (0.07)	0	1	0	0	0
Renal failure^b^	2 (0.15)	2	0	0	0	0
Investigations						
Blood glucose increased	1 (0.07)	0	1	0	0	0
Blood pressure decreased	0	0	0	1 (0.07)	1	0

^a^Multiple occurrences of the same ADR in the same patient are counted once

^b^Unexpected ADR

*ADR* adverse drug reaction

Eight serious ADRs were reported in eight pediatric patients and 13 serious ADRs were reported in 12 adult patients (Table 2). Serious ADRs reported in pediatric patients were acute kidney injury (four events), renal failure (two events), hypoxia (one event), and pneumothorax (one event). In adults, serious ADRs included pulmonary hypertension (five events), pulmonary arterial hypertension (two events), hypotension (one event), acute respiratory distress syndrome (one event), pulmonary alveolar hemorrhage (one event), pulmonary hypertensive crisis (one event), acute kidney injury (one event), and decreased blood pressure (one event). Overall, ADRs related to pulmonary hypertension (pulmonary hypertension, pulmonary arterial hypertension, and pulmonary hypertensive crisis) and ADRs related to renal disorder (acute kidney injury and renal failure) accounted for 71.43% of all serious ADRs (i.e., 15/21 events).

All serious ADRs related to pulmonary hypertension were observed in adults (eight events in seven patients), and all occurred after the completion/withdrawal of INOflo treatment. In six of eight events, pulmonary hypertension recurred even though the patients had been weaned off INOflo in a stepwise manner, in line with approved labelling [10]. After the recurrence of pulmonary hypertension, INOflo was readministered in seven of eight events; six events were subsequently resolved or resolving. The outcomes of the two remaining pulmonary hypertension–related serious ADRs were unknown due to transfer to another hospital (one event), and death due to concurrent multi-organ failure (one event).

Seven serious ADRs related to renal disorder occurred in seven patients; of these, six events occurred in pediatrics. In two of seven events, acute kidney injury occurred as a complication before INOflo treatment. In five of seven events, INOflo was continued for 2–6 days after the onset of the renal event. In four of seven events, serious ADRs related to renal disorder were resolved or resolving with treatment such as peritoneal dialysis. The outcomes of the three remaining renal disorder–related serious ADRs were not recovered (two events) and death due to exacerbation of concurrent pulmonary edema (one event).

When ADRs in pediatric patients were assessed by baseline characteristics, no background factors were significantly associated with the incidence of ADRs (see Supplementary Table S1; Online Resource 1). Similarly, the initial INOflo dose received and duration of INOflo treatment had no significant impact on the incidence of ADRs in pediatric patients (Supplementary Table S1; Online Resource 1).

When ADRs in adults were assessed by patient characteristics, there was a statistically significant relationship between baseline Pp/Ps and the incidence of ADRs (Supplementary Table S1; Online Resource 1). The incidence of ADRs was 0% in adults with a baseline Pp/Ps of ≤ 0.2, and increased to 8.33% as baseline Pp/Ps increased to > 0.8 to ≤ 1.0 No other baseline characteristics had a statistically significant impact on the incidence of ADRs. The initial INOflo dose received and the duration of INOflo treatment also had no significant impact on the incidence of ADRs in adults (Supplementary Table S1; Online Resource).

**Fig. 2:**
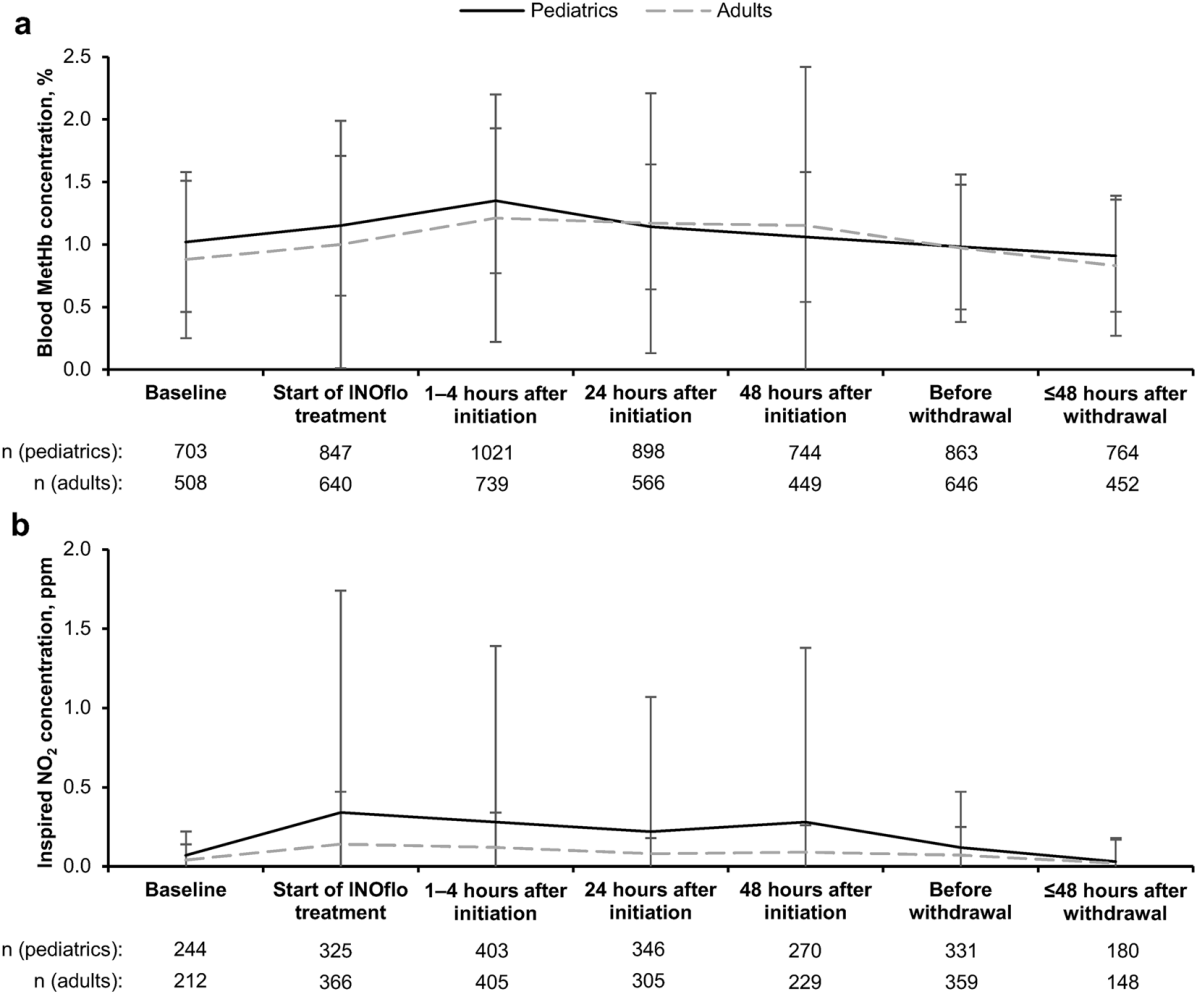
Mean (**a**) blood MetHb concentration and (**b**) inspired NO_2_ concentration over time in pediatric and adult patients who received INOflo for pulmonary hypertension during the perioperative period of cardiac surgery (safety analysis population) Analyses are based on observed data, with no imputation for missing values. Error bars present SD. *MetHb* methemoglobin, *NO*_*2*_ nitrogen dioxide, *SD* standard deviation

The mean ± SD blood MetHb concentration at baseline was 1.0 ± 0.6% in pediatrics and 0.9 ± 0.6% in adults, and transiently increased to 1.4 ± 0.6% and 1.2 ± 1.0%, respectively, at 1–4 h after INOflo initiation (Fig. 2a). In both pediatrics and adults, blood MetHb concentrations returned to baseline levels ≤ 48 h after completion/withdrawal of INOflo treatment. Three methemoglobinemia ADRs were reported in three patients (two pediatric patients [0.15%] and one adult patient [0.07%]; Table 2); all three events were non-serious, and the peak blood MetHb concentration in these three patients did not exceed 1.4%. In both pediatric and adult patients, mean inspired NO_2_ concentrations did not exceed 0.5 ppm at any time during the observation period (Fig. 2b).

### Effectiveness outcomes

**Fig. 3:**
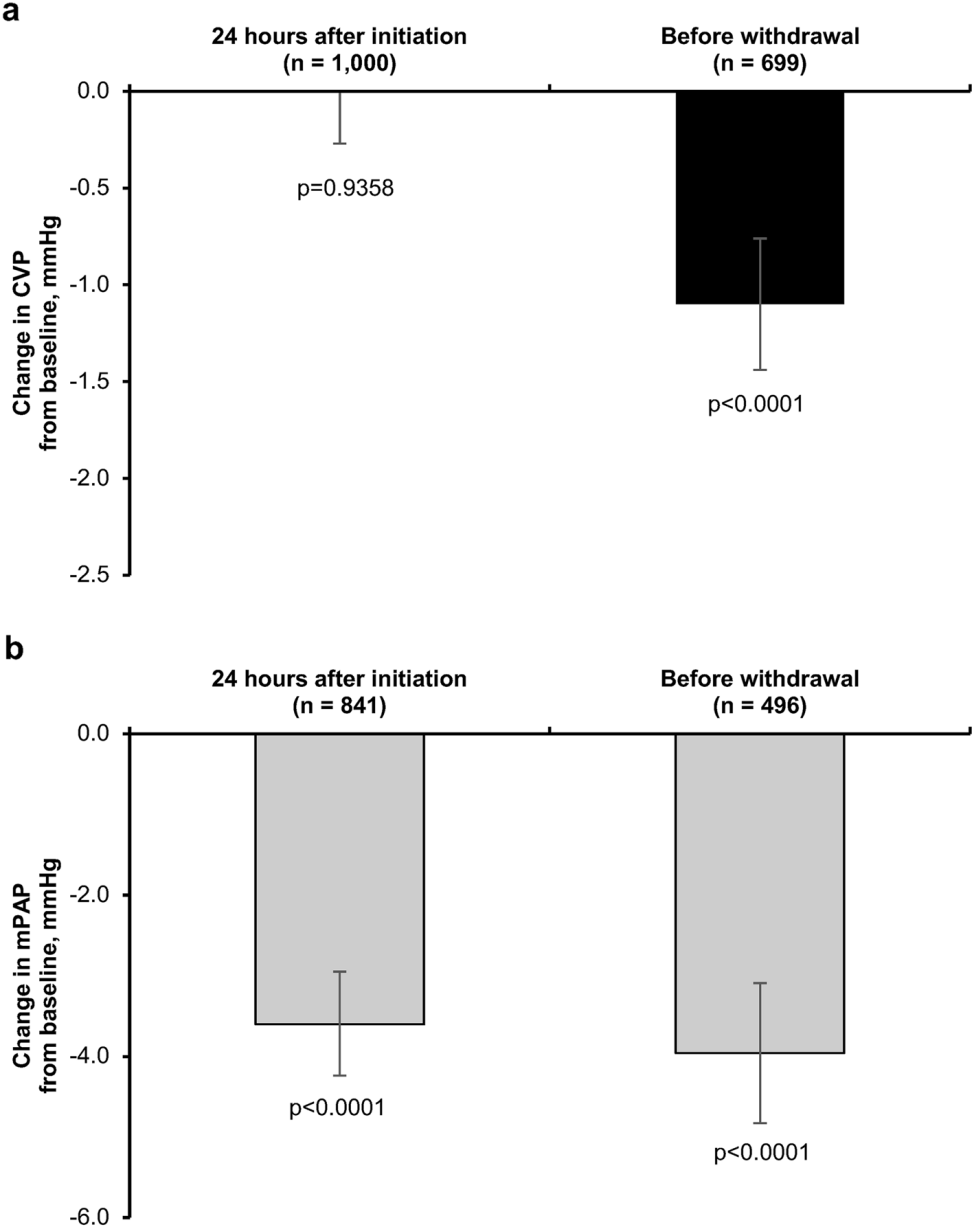
Mean change in (**a**) CVP among pediatrics and (**b**) mPAP among adults who received INOflo for pulmonary hypertension during the perioperative period of cardiac surgery (effectiveness analysis population) Analyses are based on observed data, with no imputation for missing values. Error bars present 95% CI; p-values were calculated using paired t-tests. *CI* confidence interval, *CVP* central venous pressure, *mPAP* mean pulmonary arterial pressure

With respect to the primary effectiveness endpoints, INOflo treatment was associated with significant reductions in CVP among pediatrics and mPAP among adults (Fig. 3). At baseline, mean ± SD CVP was 10.0 ± 4.5 mmHg in pediatrics (n = 1038) and 12.0 ± 5.2 mmHg in adults (n = 1102). In pediatric patients, the mean change in CVP from baseline was − 0.01 mmHg (95% CI  − 0.27, 0.25; n = 1,000) at 24 h after INOflo initiation and − 1.10 mmHg (95% CI  − 1.44,  − 0.76; n = 699) at the completion/withdrawal of treatment (Fig. 3a). For reference, corresponding mean changes in CVP among adult patients were − 0.83 mmHg (95% CI − 1.14, − 0.52; n = 1024) and − 1.60 mmHg (95% CI − 2.02, − 1.17; n = 696), respectively (Supplementary Fig. S1a; Online Resource 1).

At baseline, mean ± SD mPAP was 26.8 ± 9.8 mmHg in adults (n = 951) and 22.1 ± 12.6 mmHg in pediatrics (n = 221). In adult patients, the mean change in mPAP from baseline was − 3.60 mmHg (95% CI − 4.24, − 2.95; n = 841) at 24 h after INOflo initiation, and −  3.96 mmHg (95% CI−  4.83,  − 3.09; n = 496) at the completion/withdrawal of treatment (Fig. 3b). For reference, corresponding mean changes from baseline in mPAP among pediatrics were − 2.24 mmHg (95% CI − 3.50,  − 0.97; n = 102) and − 2.68 mmHg (95% CI −  4.31, − 1.05; n = 69), respectively (Supplementary Fig. S1b; Online Resource 1).

**Fig. 4:**
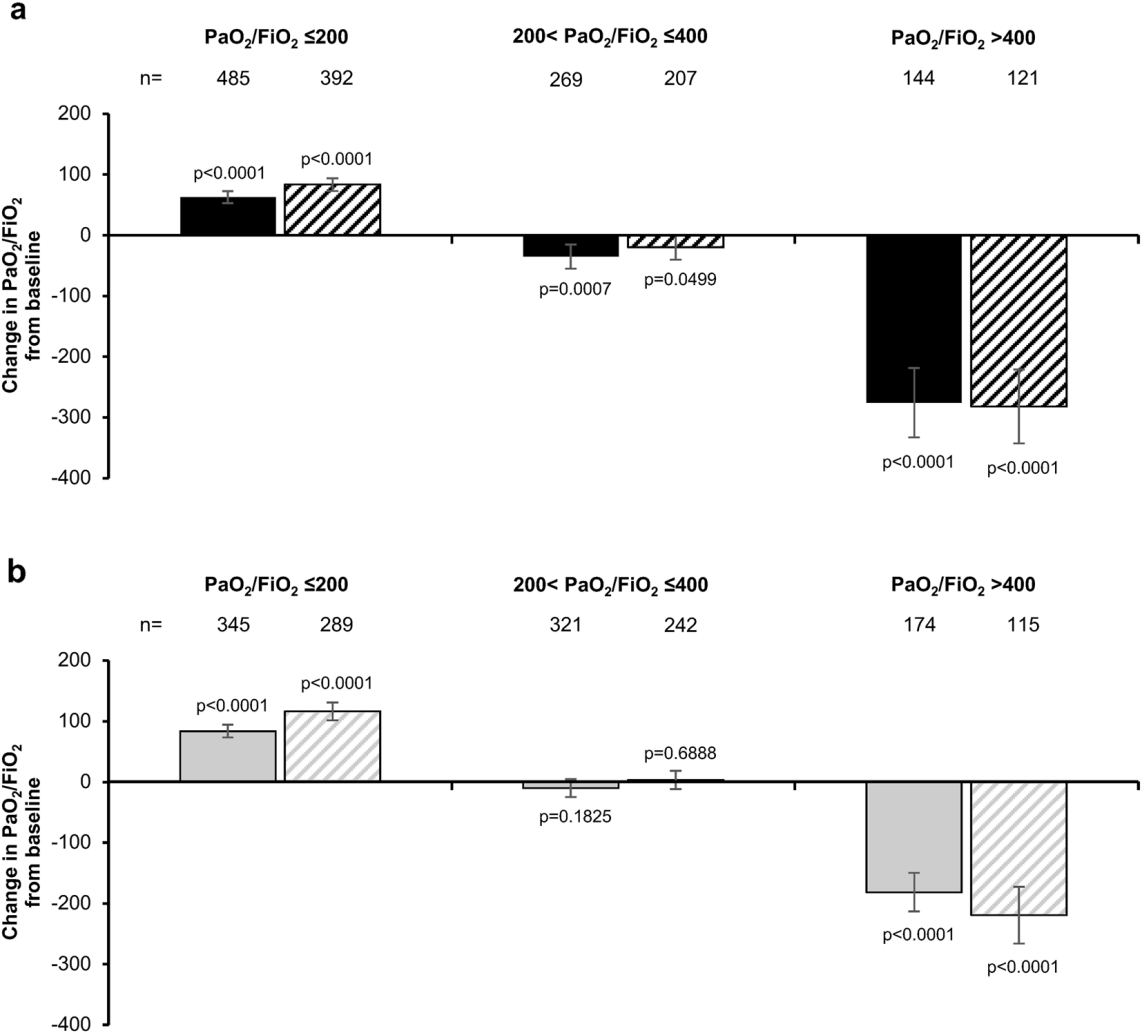
Mean change in PaO_2_/FiO_2_ ratio by baseline PaO_2_/FiO_2_ in (**a**) pediatric and (**b**) adult patients who received INOflo for pulmonary hypertension during the perioperative period of cardiac surgery (effectiveness analysis population) Solid bars present change in PaO_2_/FiO_2_ from baseline at 24 h after iINOflo initiation; striped bars present change in PaO_2_/FiO_2_ from baseline at treatment completion/withdrawal. Analyses are based on observed data, with no imputation for missing values. Error bars present 95% CI; p-values were calculated using paired t-tests *CI* confidence interval, *FiO*_*2*_ fraction of inspired oxygen, *PaO*_*2*_ partial pressure of arterial oxygen

PaO_2_/FiO_2_ was significantly increased with INOflo treatment in pediatric and adult patients with PaO_2_/FiO_2_ ≤ 200 at baseline (Fig. 4). In this subgroup, mean ± SD PaO_2_/FiO_2_ at baseline was 92.6 ± 51.7 among pediatrics (n = 527) and 116.2 ± 42.8 among adults (n = 366). In pediatric patients with PaO_2_/FiO_2_ ≤ 200 at baseline, mean change in PaO_2_/FiO_2_ from baseline was + 62.4 (95% CI 52.20, 72.59; n = 485) at 24 h after INOflo initiation and + 83.3 (95% CI 72.60, 94.06; n = 392) at the completion/withdrawal of treatment (Fig. 4a). In adults with PaO_2_/FiO_2_ ≤ 200 at baseline, corresponding mean changes in PaO_2_/FiO_2_ from baseline were + 83.8 (95% CI 73.01, 94.55; n = 345) and + 116.1 (95% CI 101.37, 130.83; n = 289), respectively (Fig. 4b).

With respect to secondary effectiveness endpoints, INOflo treatment in pediatric patients was associated with significant changes from baseline in mSBP and SpO_2_ at 24 h after INOflo initiation and at the completion/withdrawal of treatment (Supplementary Fig. S2; Online Resource 1). At baseline, mean ± SD mSBP among pediatrics was 54.9 ± 12.8 mmHg (n = 1271) and mean ± SD SpO_2_ was 90.7 ± 11.2% (n = 1154). At 24 h after INOflo initiation, the mean change in mSBP from baseline was + 5.9 mmHg (95% CI 5.20, 6.77; n = 1232), which increased to + 7.3 mmHg (95% CI 6.31, 8.19; n = 999) at the completion/withdrawal of treatment (Supplementary Fig. S2a; Online Resource 1). The mean change in SpO_2_ at the corresponding time points were + 2.2% (95% CI 1.56, 2.82; n = 1077) and + 2.2% (95% CI 1.48, 2.91; n = 919), respectively (Supplementary Fig. S2b; Online Resource 1). PCWP, pulmonary vascular resistance, and cardiac output were not assessed in pediatrics due to the limited number of patients with data available for analysis.

In adults, significant changes from baseline were observed in mSBP and PCWP at both time points, and in cardiac output at 24 h after INOflo initiation (Supplementary Fig. S3; Online Resource 1). At baseline, mean ± SD mSBP among adults was 70.5 ± 14.7 mmHg (n = 1285), mean ± SD SpO_2_ was 97.8 ± 3.4% (n = 1036), mean ± SD cardiac output was 3.8 ± 2.2 L/min (n = 522), and mean ± SD PCWP was 18.2 ± 9.3 mmHg (n = 223). Mean change in mSBP from baseline was + 2.1 mmHg (95% CI 1.10, 3.02; n = 1217) at 24 h after INOflo initiation, and + 3.4 mmHg (95% CI 2.15, 4.59; n = 896) at the completion/withdrawal of treatment (Supplementary Fig. S3a; Online Resource 1). There were no statistically significant changes in SpO_2_ levels from baseline to 24 h after INOflo initiation (mean change + 0.3% [95% CI 0.00, 0.50]; n = 957) or till treatment completion/withdrawal (mean change 0.3% [95% CI −  0.03, 0.59]; n = 719) (Supplementary Fig. 3b; Online Resource 1). The mean change in cardiac output was statistically significant at 24 h after INOflo initiation (mean change + 0.2 L/min [95% CI 0.03, 0.41]; n = 433), but not at the completion/withdrawal of treatment (mean change + 0.2 L/min [95% CI − 0.07, 0.46]; n = 242; Supplementary Fig. 3c; Online Resource 1). PCWP was significantly reduced at 24 h after INOflo initiation (mean change −  1.9 mmHg [95% CI − 3.06, − 0.70]; n = 125) and at the completion/withdrawal of treatment (mean change − 3.7 mmHg [95% CI − 5.63, −  1.71]; n = 60; Supplementary Fig. 3d; Online Resource 1). Pulmonary vascular resistance was not assessed in adults due to the limited number of patients with data available for analysis.

Regarding overall effectiveness, the proportion of patients deemed “mildly improved” or better after INOflo treatment was 92.2% in pediatrics (1,236 of 1,340 patients) and 83.1% in adults (1,189 of 1,431 patients). When INOflo effectiveness was assessed by baseline patient characteristics, the timing of INOflo treatment was a significant factor among pediatrics but not among adults. In pediatric patients, INOflo was most effective among those who received it during cardiac surgery (95.4%) than in those who received INOflo during and after (92.9%) or after (91.0%) cardiac surgery (p = 0.0189). In adult patients, there was no significant difference in treatment effectiveness between patients who received INOflo during (81.8%), during and after (82.9%), or after (83.2%) cardiac surgery (p = 0.8245).

## Discussion

This post-marketing study of 2,817 patients represents the largest survey of INOflo for the perioperative treatment of pulmonary hypertension during cardiac surgery in Japan. Consistent with the previous Japanese phase 3 study [10], perioperative INOflo treatment was found to improve hemodynamics and oxygenation while demonstrating an acceptable safety profile in both pediatric and adult patients. These data further support the use of INOflo for the perioperative management of pulmonary hypertension in Japanese clinical practice.

Data from this study provide an insight into the demographic and clinical profile of patients receiving INOflo for its approved indication in Japan. At baseline, approximately 70% of pediatric patients were aged < 1 year, which included 21% of patients who received INOflo during the neonatal period. This subgroup may comprise newborns with severe congenital heart disease and who required cardiac surgery in the early postnatal period [14, 15]. Among adults, 57% of patients who received INOflo were aged ≥ 65 years, suggesting that in an aging population, cardiac surgeries are increasingly performed on patients traditionally considered to be unsuitable candidates [16, 17]. In both pediatrics and adults, INOflo was most often administered after cardiac surgery (59% and 58% of patients, respectively), indicating that hemodynamic stability at bypass withdrawal does not discount the possibility of postoperative instability. Finally, it was interesting to note that while many patients who received INOflo had CVP < 15 mmHg and/or mPAP < 25 mmHg at baseline, a considerable proportion had Pp/Ps > 0.4. Thus, although CVP and mPAP may appear to be stable at low levels, the ratio of pulmonary to SBP may be imbalanced, in which case Pp/Ps may be an important consideration in iNO treatment decisions. In the most recent European Society of Cardiology and European Respiratory Society (ESC/ERS) guidelines for the management of pulmonary hypertension, the mPAP threshold used to define pulmonary hypertension was reduced from > 25 to > 20 mm Hg [18]. The approved indication for INOflo does not specify a threshold or definition for pulmonary hypertension [10]; however, our data suggest that diagnosis and treatment should be based on multiple hemodynamic parameters, including Pp/Ps.

INOflo was generally well tolerated in this study; ADRs were reported in 32 of 2817 patients overall and no single ADR had an incidence rate > 1%. Pp/Ps has previously been identified as a predictor of hemodynamic complications and morbidity after cardiac surgery [19, 20] and is used as an index for pediatric heart surgery [21]; indeed, when we examined the relationships between baseline patient characteristics and the incidence of ADRs in this study, we found that increasing baseline Pp/Ps in adults was significantly associated with increasing ADR incidence. No other baseline characteristic, and neither the initial INOflo dose received nor the duration of INOflo treatment, had a significant impact on the incidence of ADRs in pediatric and adult patients.

Almost 40% of serious ADRs reported in this study were events related to pulmonary hypertension (8/21 serious ADRs), which were events not commonly observed in previous INOflo studies [10]. In this study, serious ADRs related to pulmonary hypertension occurred in adults after the completion/withdrawal of INOflo treatment, and generally resolved upon retreatment with INOflo, suggesting rebound pulmonary hypertension. Rebound pulmonary hypertension after iNO withdrawal has previously been observed with INOflo, and is in part attributed to the inhibition of endogenous NO synthesis by exogenous iNO [22, 23]. This phenomenon highlights the importance of close monitoring when weaning patients off iNO therapy.

Acute kidney injury is a serious postoperative complication of cardiac surgery, with an estimated incidence rate of 22% among adults [24]. In this study, one-third of serious ADRs were renal disorder–related events (7/21 serious ADRs), and acute kidney injury was the most frequent renal disorder–related serious ADR (five events in four pediatrics [0.3%] and one adult [0.1%]). Although a previous systematic review reported a significant increase in the risk of renal failure with iNO in patients with acute respiratory distress syndrome [25], another meta-analysis found a reduced risk of acute kidney injury with iNO among patients undergoing cardiac surgery [26]. Acute kidney injury events were not commonly observed in previous INOflo studies [10] and current Japanese clinical practice guidelines do not consider iNO therapy during cardiac surgery as a risk factor for acute kidney injury [27]; however, physicians should be aware of the potential risk for this event when administering perioperative iNO therapy.

The oxidation of hemoglobin to MetHb is a safety consideration of iNO therapy [28], and blood MetHb concentrations above 10–20% are of clinical concern [29]. In both pediatric and adult patients, mean blood MetHb concentrations showed transient increases to ≤ 1.4% at 1–4 h after INOflo initiation and returned to baseline levels within 48 h of completion/withdrawal of treatment. The incidence of methemoglobinemia ADRs (three non-serious events in two pediatrics [0.1%] and one adult [0.1%]) was no higher than previous iNO studies [10, 30], and peak blood MetHb concentrations in patients with methemoglobinemia (≤ 1.4%) remained within the normal physiological range of 0–2% [29].

iNO can react with oxygen to produce NO_2_, and the National Institute for Occupational Safety and Health recommends a NO_2_ short-term exposure limit of 1 ppm [31]. In this study, mean inspired NO_2_ concentrations remained ≤ 0.5 ppm throughout the observation period for both pediatric and adult patients, indicating that clinically significant NO_2_ exposure did not occur.

Our study also demonstrated the hemodynamic and oxygenation improvements associated with INOflo treatment for pulmonary hypertension during cardiac surgery. With respect to the primary effectiveness endpoints, we observed a significant reduction in CVP after the completion/withdrawal of INOflo treatment among pediatric patients, and significant reductions in mPAP at 24 h after INOflo initiation and after completion/withdrawal of treatment among adult patients. This may be the result of iNO selectively relaxing smooth muscle in pulmonary vessels [32]. These results are broadly consistent with the previous phase 3 trial of perioperative INOflo for pulmonary hypertension in Japan, which reported improved mean CVP and PaO_2_/FiO_2_ in pediatrics, and reduced mPAP in adults [10]. Because this study included relatively less severe cases than the phase 3 trial, differences between the two studies in 24-h hemodynamic outcomes associated with INOflo treatment may be explained by differences in baseline hemodynamics.

In pediatric and adult patients with PaO_2_/FiO_2_ ≤ 200 at baseline (i.e., Berlin-defined moderate-to-severe respiratory failure [33]), INOflo treatment was additionally associated with significant improvements in PaO_2_/FiO_2_ at both time points. This may, in part, be due to a reduced incidence of ventilation perfusion mismatches and/or intrapulmonary shunts, resulting in more efficient oxygenation. Our study included a subset of patients with PaO_2_/FiO_2_ > 400 at baseline, who may have been connected to some form of extracorporeal circulation (i.e., CPB or extracorporeal membrane oxygenation [ECMO]). Although a significant reduction in PaO_2_/FiO_2_ was observed after INOflo initiation in patients with PaO_2_/FiO_2_ > 400 at baseline, mean PaO_2_/FiO_2_ at INOflo withdrawal was ≥ 300 in this subgroup (data not shown), which is considered to be a satisfactory value for perioperative hemodynamics.

In the Japanese phase 3 trial of perioperative INOflo treatment for pulmonary hypertension, eligible pediatric patients underwent Glenn surgery, Fontan surgery, or other procedures for congenital heart disease, while adult patients were limited to those receiving left ventricular assist device implantation [10, 12]. In comparison, our study included pediatric and adult patients who underwent a variety of procedures, including cardiac transplantation, valve repair, aorta replacement, coronary artery bypass graft, atrial or ventricular septal defect closure, and tetralogy of Fallot repair. It should also be noted that there may be differences in perioperative management practices between clinical sites in this study, such as the concomitant use of intra-aortic balloon pumps, ECMO, or mechanical circulatory support. This suggests that the results of this post-marketing study may be more generalizable to patients receiving INOflo for its approved indication than the findings of previous clinical trials.

This post-marketing study was limited by its short observation period and lack of control group to compare safety and effectiveness. The study findings may have been influenced by confounding factors, as it included patients undergoing a wide range of cardiac procedures, and clinical sites that may differ with respect to perioperative management practices and the use of anesthetics and other drugs. In addition, the study protocol did not provide a prescribed method of measuring the effectiveness endpoints, and effectiveness data were missing for many patients. For example, Swan-Ganz catheterization was not conducted in some patients, especially those undergoing emergency procedures including type A aortic dissection surgery. Furthermore, single- and biventricular repair procedures were grouped together in pediatric patients, but as their cardiac morphology and circulatory dynamics are markedly different, it may be more desirable to analyze and investigate these procedures separately.

## Conclusion

This post-marketing study of 2,817 pediatric and adult patients confirmed the real-world safety and effectiveness of INOflo for the improvement of perioperative pulmonary hypertension associated with cardiac surgery in Japan. In this study, perioperative INOflo treatment for pulmonary hypertension was generally well tolerated among pediatric and adult patients, and no new safety signals were identified. In terms of effectiveness, INOflo treatment was associated with improvements in hemodynamics and oxygenation compared with baseline in both pediatric and adult patients. Future studies may seek to further investigate patient factors that predict clinical outcomes with INOflo treatment; nevertheless, current data support the use of INOflo for the perioperative management of pulmonary hypertension in Japanese clinical practice.

### Supplementary Information

Below is the link to the electronic supplementary material.

Supplementary file1 (PDF 460 kb)

## Data Availability

Data generated/analyzed during the current study are available from the corresponding author upon reasonable request.

## Abbreviations

ADR: Adverse drug reaction
CI: Confidence interval
CPB: Cardiopulmonary bypass
CRF: Case report form
CVP: Central venous pressure
ECMO: Extracorporeal membrane oxygenation
iNO: Inhaled nitric oxide
MedDRA: Medical dictionary for regulatory activities terminology
MetHb: Blood methemoglobin
mPAP: Mean pulmonary arterial pressure
mSBP: Mean systemic blood pressure
NO: Nitric oxide
NO_2_: Nitrogen dioxide
PaO_2_/FiO_2_: Partial pressure of arterial oxygen/fraction of inspired oxygen ratio
PCWP: Pulmonary capillary wedge pressure
ppm: Parts per million
Pp/Ps: Mean pulmonary arterial pressure/mean systemic blood pressure
SBP: Systemic blood pressure
SD: Standard deviation
SpO_2_: Percutaneous arterial oxygen saturation
