# Supplementary material for: Real-world safety and effectiveness of inhaled nitric oxide therapy for pulmonary hypertension during the perioperative period of cardiac surgery: a post-marketing study of 2817 patients in Japan
Source: Gen Thorac Cardiovasc Surg. 2023 Sep 15;72(5):311–23. doi: 10.1007/s11748-023-01971-2 (PMC11018662; doi:10.1007/s11748-023-01971-2)
Supplement: Supplementary file 1 — Supplementary file1 (PDF 460 kb) [file 11748_2023_1971_MOESM1_ESM.pdf]

Real-world safety and effectiveness of inhaled nitric oxide therapy for pulmonary hypertension during the perioperative period of cardiac surgery: a post-marketing study of 2,817 patients in Japan

General Thoracic and Cardiovascular Surgery

Supplementary Material

Authors: Emi Matsugi[1], BPharm; Shigeki Takashima[2], MPharm; Shuhei Doteguchi[1], MPharm; Tomomi Kobayashi[1], MPharm; Motohiro Okayasu[1], PhD

Affiliations:[1]Medical Affairs, Mallinckrodt Pharmaceuticals, Tokyo, Japan; [2]Pharmacovigilance, Mallinckrodt Pharmaceuticals, Tokyo, Japan.

Correspondence: Emi Matsugi

Mallinckrodt Pharmaceuticals, 1-12-32 Akasaka, Minato-ku Tokyo, 107-6030, Japan.

Email: emi.matsugi@mnk.com

Table S1 Incidence of adverse drug reactions by patient baseline characteristics (safety analysis population)

	Pediatrics (n = 1,375)			Adults (n = 1,442)		
	**n**	**Patients with ≥1 ADR, n (%)[a]**	**p-value[b]**	**n**	**Patients with ≥1 ADR, n (%)[a]**	**p-value[b]**
Total ADRs	1,375	14 (1.02)	–	1,442	18 (1.25)	–
Sex						
Male	771	6 (0.78)	0.4186	861	11 (1.28)	1.0000
Female	604	8 (1.32)		580	7 (1.21)	
Age						
≤28 days	295	3 (1.02)	0.6690	–	–	0.4662
>28 days to <1 year	670	7 (1.04)		–	–	
≥1 to <5 years	348	3 (0.86)		–	–	
≥5 to < 10 years	26	1 (3.85)		–	–	
≥10 to <15 years	23	0		–	–	
≥15 to <25 years	–	–		41	0	
≥25 to <35 years	–	–		69	0	
≥35 to <45 years	–	–		104	2 (1.92)	
≥45 to <55 years	–	–		161	4 (2.48)	
≥55 to <65 years	–	–		242	4 (1.65)	
≥65 years	–	–		824	8 (0.97)	
Unknown	13	0		1	0	
Presence of pulmonary hypoplasia or history of severe lung disorder						
Yes	68	0	1.0000	80	1 (1.25)	1.0000
No	1,280	12 (0.94)		1,332	17 (1.28)	
Unknown	27	2 (7.41)		30	0	
Timing of INOflo treatment in relation to cardiac surgery						
Before	20	0	0.9994	9	1 (11.11)	0.1049
During	368	4 (1.09)		347	4 (1.15)	
After	818	8 (0.98)		840	13 (1.55)	
Before and during	2	0		0	0	
Before and after	7	0		3	0	
During and after	113	1 (0.88)		211	0	
Before, during, and after	3	0		2	0	
Other	15	0		13	0	
Unknown	29	1 (3.45)		17	0	
Presence of complications						
Yes	472	7 (1.48)	0.2591	506	10 (1.98)	0.0821
No	903	7 (0.78)		936	8 (0.85)	
Presence of concomitant drugs						
Yes	1,292	14 (1.08)	1.0000	1,321	18 (1.36)	0.3916
No	83	0		121	0	
Baseline mPAP						
<25 mmHg	160	0	0.3383	406	2 (0.49)	0.1588

≥25 to <50 mmHg	51	1 (1.96)		522	11 (2.11)	
≥50 to <75 mmHg	10	0		26	1 (3.85)	
≥75 mmHg	1	0		1	0	
Unknown	1,153	13 (1.13)		487	4 (0.82)	
Baseline CVP						
<15 mmHg	896	8 (0.89)	0.6678	785	10 (1.27)	0.5797
≥15 mmHg	172	2 (1.16)		324	6 (1.85)	
Unknown	307	4 (1.30)		333	2 (0.60)	
Baseline PaO_2/FiO_2 ratio						
≤200	542	9 (1.66)		374	9 (2.41)	
>200 to ≤400	306	4 (1.31)	0.5843	365	6 (1.64)	0.2284
>400	167	1 (0.60)		206	1 (0.49)	
Unknown	360	0		497	2 (0.40)	
Baseline Pp/Ps ratio						
≤0.2	30	0		67	0	
>0.2 to ≤0.4	101	0		461	4 (0.87)	
>0.4 to ≤0.6	46	0	0.0966	277	4 (1.44)	**0.0066**
>0.6 to ≤0.8	21	1 (4.76)		65	4 (6.15)	
>0.8 to ≤1.0	14	0		12	1 (8.33)	
>1.0	4	0		7	0	
Unknown	1,159	13 (1.12)		553	5 (0.90)	
Baseline NO concentration						
<5 ppm	16	0		–	–	
5 ppm	86	1 (1.16)		–	–	
>5 to <10 ppm	7	0		–	–	
10 ppm	532	6 (1.13)	0.7378	–	–	
>10 to <20 ppm	42	0		–	–	
20 ppm	666	6 (0.90)		–	–	
>20 ppm	22	1 (4.55)		–	–	
<10 ppm	–	–		72	2 (2.78)	
10 ppm	–	–		267	2 (0.75)	
>10 to <20 ppm	–	–		32	0	
20 ppm	–	–		951	14 (1.47)	
>20 to <30 ppm	–	–		5	0	0.8100
30 ppm	–	–		58	0	
>30 to <40 ppm	–	–		0	0	
40 ppm	–	–		24	0	
>40 ppm	–	–		2	0	
Unknown	4	0		31	0	
Type of cardiac surgery						
Placement of VAD	16	0		181	2 (1.10)	
Bidirectional Glenn surgery	135	2 (1.48)		2	0	
Fontan procedure	139	1 (0.72)		17	0	
VSD closure	183	0	0.7656	7	0	0.9992
ASD closure	20	0		7	0	
AVSD surgery	47	0		0	0	
CoA surgery	19	1 (5.26)		0	0	

TAPVR repair	91	1 (1.10)	1	0
TGA surgery	78	1 (1.28)	0	0
TOF repair	44	0	4	0
Cardiac transplant	3	0	25	0
Combine surgery	228	4 (1.75)	53	1 (1.89)
Other	354	4 (1.13)	1,072	15 (1.40)
Unknown	18	0	73	0

[a]Multiple occurrences of the same ADR in the same patient are counted once.
[b]Calculated using Fisher's exact test; significant p-values are indicated in bold.
ADR adverse drug reaction, *ASD* atrial septal defect, *AVSD* atrioventricular septal defect, *CoA* coarctation of aorta, *CVP* central venous pressure, FiO_2 fraction of inspired oxygen, *mPAP* mean pulmonary arterial pressure, *NO* nitric oxide, PaO_2 partial pressure of arterial oxygen, *Pp/Ps* mean pulmonary arterial pressure/mean systemic blood pressure, *TAPVR* total anomalous pulmonary venous connection, *TGA* transposition of the great arteries, *TOF* tetralogy of Fallot, *VAD* ventricular assist device, *VSD* ventricular septal defect.

Supplementary Fig. S1 Mean change in (a) CVP among adults and (b) mPAP among pediatrics who received INOflo for pulmonary hypertension during the perioperative period of cardiac surgery (effectiveness analysis population)

Analyses are based on observed data, with no imputation for missing values. Error bars present 95% CI; p-values were calculated using paired t-tests.

CI confidence interval, *CVP* central venous pressure, *mPAP* mean pulmonary arterial pressure.

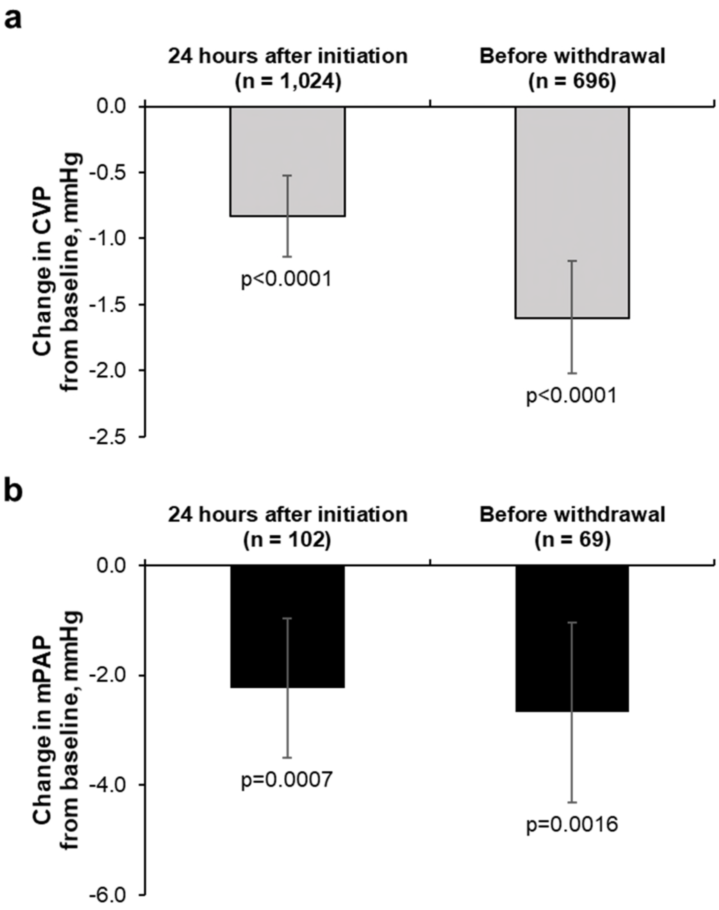

Supplementary Fig. S2 Mean change in (a) mSBP and (b) SpO_2 in pediatric patients who received INOflo for pulmonary hypertension during the perioperative period of cardiac surgery (effectiveness analysis population).

Analyses are based on observed data, with no imputation for missing values. Error bars present 95% CI; p-values were calculated using paired t-tests.

CI confidence interval; *mSBP* mean systemic blood pressure, *SpO₂* percutaneous arterial oxygen saturation.

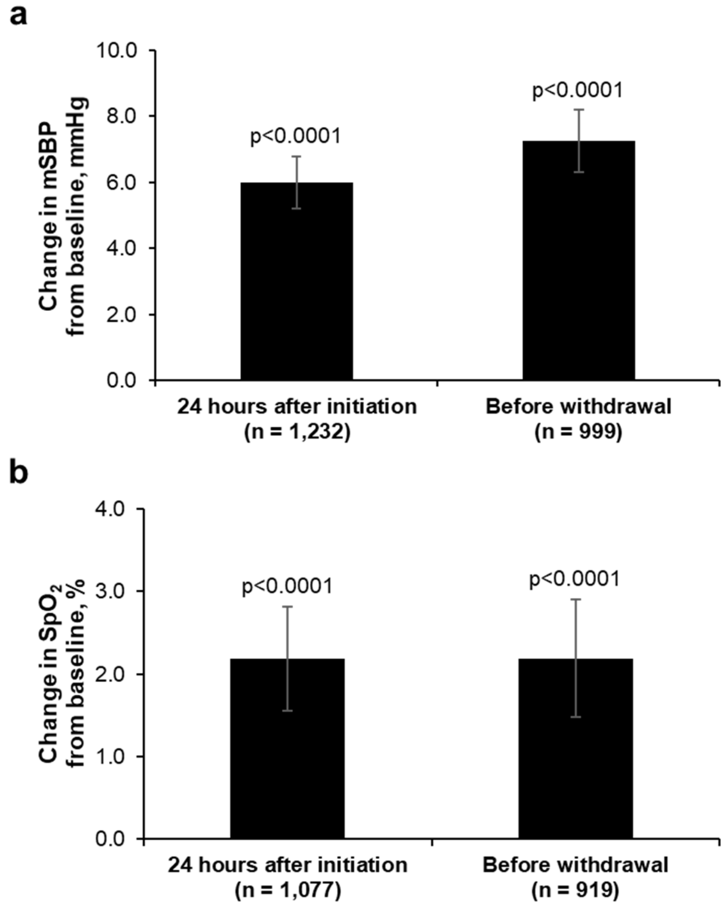

Supplementary Fig. S3 Mean change in (a) mSBP, (b) SpO_2, (c) cardiac output, and (d) PCWP in adult patients who received INOflo for pulmonary hypertension during the perioperative period of cardiac surgery (effectiveness analysis population).

Analyses are based on observed data, with no imputation for missing values. Error bars present 95% CI; p-values were calculated using paired t-tests.

CI confidence interval, *mSBP* mean systemic blood pressure, *PCWP* pulmonary capillary wedge pressure, *SpO_2* percutaneous arterial oxygen saturation.

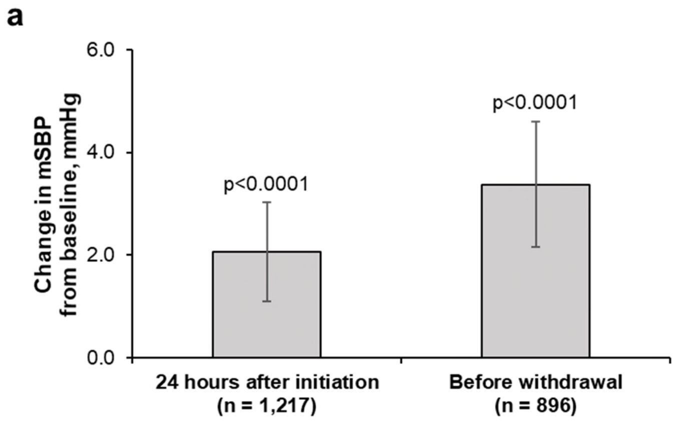

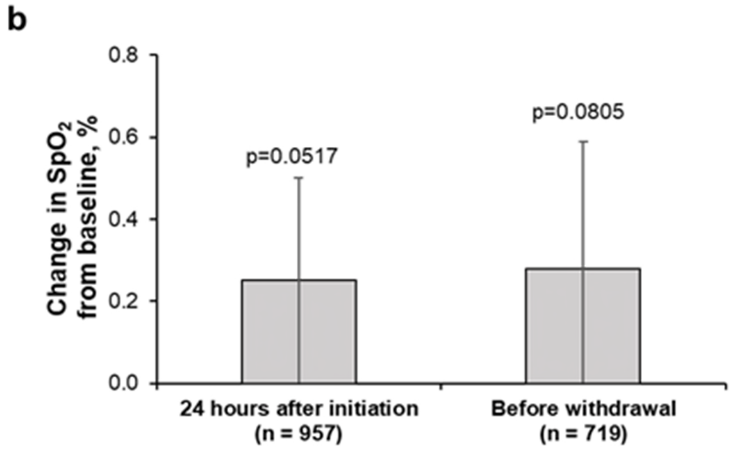

c

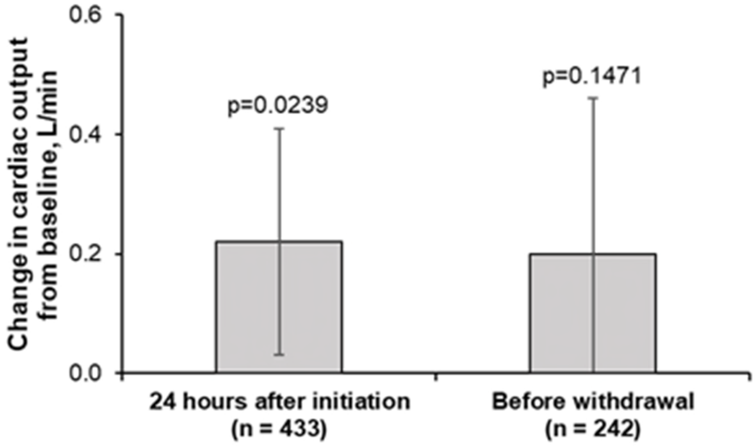
Change in cardiac output from baseline, L/min
0.6
0.4
0.2
0.0
p=0.0239
p=0.1471
24 hours after initiation (n = 433)
Before withdrawal (n = 242)

d

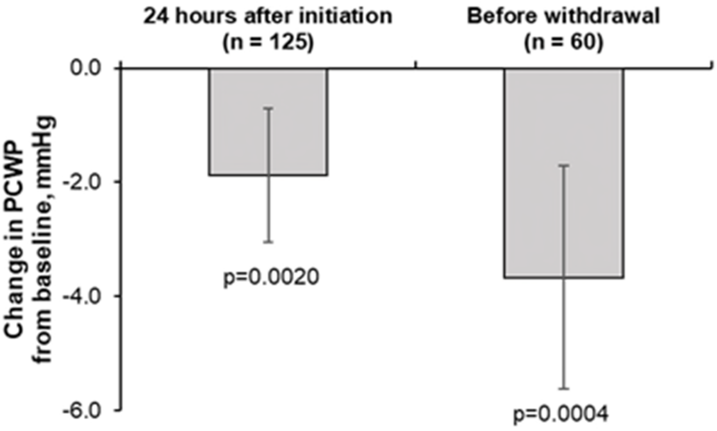
24 hours after initiation (n = 125)
Before withdrawal (n = 60)
Change in PCWP from baseline, mmHg
0.0
-2.0
-4.0
-6.0
p=0.0020
p=0.0004
